# Nutritional quality profiles of six microgreens

**DOI:** 10.1038/s41598-025-85860-z

**Published:** 2025-02-20

**Authors:** Sibel Balik, Farah Elgudayem, Hayriye Yildiz Dasgan, Nesibe Ebru Kafkas, Nazim S. Gruda

**Affiliations:** 1 Department of Horticulture, Faculty of Agriculture, University of Cukurova, 01330 Adana, Turkey; 2 Laboratory of Ecosystems and Biodiversity in Arid areas in Tunisia, Department of Life Sciences, Faculty of Sciences of Sfax, University of Sfax, 3000 Sfax, Tunisia; 3 Institute of Crop Science and Resource Conservation, Division of Horticultural Sciences, University of Bonn, Bonn, Germany

**Keywords:** Antioxidant, Functional food, Immature greens, Nutritional comparison, Bioactive compounds, Phytonutrients, Plant sciences, Environmental sciences

## Abstract

Globally, one in every four individuals faces a deficiency in essential micronutrients. Harvested early from various vegetables, grains, and herbs, microgreens have rich nutritional profiles that can mitigate nutrient deficiencies. Here, we analyzed six microgreens’ nutritional profiles for broccoli, black radish, red beet, pea, sunflower, and bean. Ascorbic acid content varied widely, from 32.72 mg/100 g fresh weight (FW) in red beet to 80.45 mg/100 g FW in beans. All microgreens exhibited high macro elements (mg/100 g FW), with potassium ranging from 187.07 to 416.05, magnesium from 45.96 to 86.83, calcium from 67.18 to 148.63, and phosphorus from 2.57 to 4.88. They also contained significant microelements (µg/100 g FW), including iron from 524 to 2610, manganese from 176.32 to 350.56, zinc from 31.92 to 129.78, and copper from 458.84 to 956.34. Glucose content surpassed sucrose and fructose, ranging from 0.114 to 0.580 mg/100 g FW. Among organic acids, citric acid was highest in red beet, succinic acid in beans, and fumaric acid in sunflower. Broccoli microgreens had the highest total phenolic content (825.53 mg GA/100 g FW), while beans had the highest total flavonoid content (758.0 mg RU/100 g FW). Black radish microgreens demonstrated the highest antioxidant capacity. Additionally, volatile aromatic compounds were analyzed across the six microgreen species. These findings highlight the nutritional potential of microgreens, advocating for their inclusion in diets to enhance human health. Red beet microgreens were the richest in organic acids, particularly citric acid, and flavonoids, supporting antioxidant activity, while black radish microgreens exhibited the highest DPPH antioxidant capacity and phenolic content. Bean microgreens stood out for their high ascorbic acid content. Sunflower microgreens had the highest levels of calcium and fumaric acid. Broccoli microgreens were abundant in phenolic compounds and contained high concentrations of iron and manganese. Finally, pea microgreens excelled in phosphorus and copper content.

**Supplementary Information:**

The online version contains supplementary material available at 10.1038/s41598-025-85860-z.

## Introduction

In light of projections related to global warming, it is anticipated that the total global food demand will surge by 35–56% by the year 2050^1^. However, FAO data from 2019 shows that 41.9% of the world’s population struggled to maintain a healthy diet^2^. Alarmingly, one in every four individuals worldwide faces a deficiency in essential micronutrients^3^, emphasizing the need to improve nutritional standards to promote overall well-being. Addressing these nutritional challenges necessitates comprehensive enhancements across the entire food chain, from production to consumption. Among the critical global concerns, mineral deficiency in nutrition, also known as “hidden hunger”^3^, emerges as a prominent issue^4^. Amid this, international interest in healthy dietary practices has surged, increasing the popularity of fresh, ready-to-eat foods like sprouted seeds and microgreens^5^.

Recognizing these trends highlights the nutritional potential of microgreens. These trendy greens, popular in upscale markets and restaurants, are harvested 7-14-21 days after germination, measuring 2.5 to 7.6 cm, and sold with stems and cotyledons intact. Despite their small size, they offer intense flavors, vibrant colors, and tender textures, making them a novel ingredient in salads, soups, sandwiches, and edible garnishes^6–8^.

Due to their nutrient-rich composition, microgreens are labeled functional foods and are increasingly seen as a potential resource for nutritional security^9^. Microgreens can swiftly produce nutrient-dense in limited spaces with minimal resources, offering a convenient solution for diversifying diets and addressing global malnutrition. This is especially important in regions facing fresh vegetable shortages due to climate change, emergencies, or conflicts. Additionally, microgreens are promising candidates for agronomic biofortification to enhance essential microelements content^10–12^. Today, microgreens continue to captivate consumers due to their intriguing nutritional profiles^13^.

Micronutrient deficiencies are a growing global health issue, often contributing to weakened immune responses, delayed growth, and developmental problems, particularly in vulnerable populations. Traditional food sources may not always meet dietary needs, so searching for nutrient-dense alternatives is critical. Microgreens, harvested at early growth stages, present a promising solution; however, systematic research on their exact nutritional impact and health benefits still needs to be conducted. Addressing this gap is essential to understanding how microgreens can be integrated into human diets to combat nutrient deficiencies.

This research aims to provide a comprehensive analysis of the nutritional potential of microgreens by comparing the bioactive compounds found in different species, including broccoli, black radish, red beet, pea, sunflower, and bean. We selected broccoli, black radish, red beetroot, sunflower, pea, and bean microgreens due to their popularity, widespread consumption, and biodiversity: broccoli and black radish belong to *Brassicaceae* family, red beetroot to *Amaranthaceae* (formerly classified under *Chenopodiaceae*, sunflower to *Asteraceae*, pea and bean to *Fabaceae* (also known as the *Leguminosae* family). Further, they have diverse nutritional profiles, which allowed us to evaluate different dietary parameters. The central hypothesis of this study is that microgreens, with their concentrated levels of vitamins, minerals, phenolic compounds, and antioxidants, can serve as a viable nutritional supplement. They hold the potential to effectively address nutrient deficiencies and improve overall health outcomes.

## Materials and methods

### Plant materials and growth conditions for microgreens

**Fig. 1:**
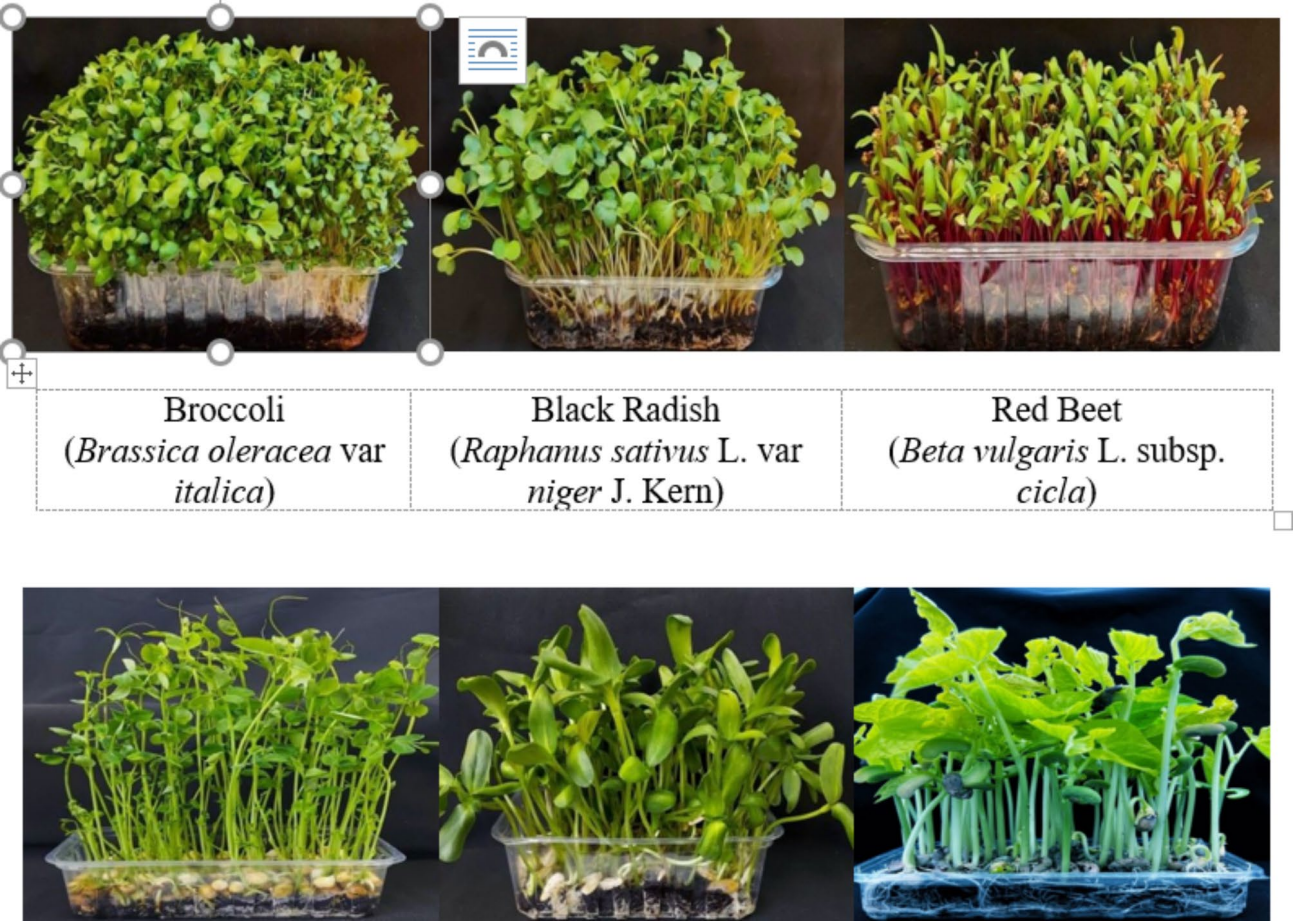
Images of microgreens from six different plant species grown in the study.

This study was conducted in a growth chamber using nutrient-rich microgreens from various plant families: black radish and broccoli (*Brassicaceae*), red beet (*Amaranthaceae*), pea and bean (*Leguminosae*), and sunflower (*Asteraceae*) (Fig. 1). Seeds were sourced from different seed companies and Cukurova University. Microgreens were grown at 20 °C ± 2 for black radish, broccoli, and red beet, and 23 °C ± 2 for peas, beans, and sunflowers, with 16 h of light and 8 h of darkness, under 60% relative humidity and a light intensity of 350 µmol/m^2^/s.

The containers made of plastic with dimensions of 16 cm x 9 cm x 7 cm (length x width x height) were used as cultivation trays. A peat-based medium was used to grow microgreens. Plants were supplied with the following nutrient solution in ¼ strength (in mg/ L): N (200), P (50), K (300), Ca (200), Mg (65), Fe (5.0), Mn (0.8), Cu (0.3), Zn (0.3), B (0.3) and Mo (0.05). Depending on the species, microgreens were harvested 7 to 21 days after germination. This time frame was when the first true leaves developed, following the initial cotyledon leaves (Fig. 1).

### Reagents and chemicals

The following reagents and chemicals were used: ultra-pure water produced in a Milli-Q system (Millipore, USA), with a resistivity of 18.2 MΩ·cm, temperature-compensated (TC) to 25 °C, sulfuric acid, hydrochloric acid, Folin-Ciocalteu, sodium carbonate, 1,1-diphenyl-2-picrylhydrazyl (DPPH), methanol, calcium chloride, salicylic acid, and sodium hydroxide were purchased from Sigma-Aldrich. Standard fructose, glucose, sucrose, gallic acid, and L-ascorbic acid were purchased from Sigma-Aldrich (St. Louis, MO, USA). Certified reference materials and highly purified analytical-grade standards and chemicals were used for the HPLC, GC-MS, and spectrophotometric analyses to ensure the accuracy and reliability of the results. Standard glucose (Purity − 99.5%), fructose, sucrose (Purity − 99.99%), L-ascorbic acid (≥ 99%), and gallic acid (98–99.5%) were purchased from Sigma- Aldrich.

### Determination of ascorbic acid content

The analysis of ascorbic acid in samples obtained from microgreens will be determined using the HPLC (Shımadzu, Prominence LC-20 A, Kyoto, Japan) technique with a UV detector and an HPX 87 H (300 × 7.8 mm, 5 μm) column, following the method developed by Bozan et al.^14^. Each plant sample (0.5 g) was mixed with 2 mL of metaphosphoric acid (3% v/v) to extract ascorbic acid. The mixture was immersed in an ultrasonic water bath for 15 min. Then, it was centrifuged for 15 min at 5500 rpm, and the supernatant was filtered with 0.45 μm Whatman nylon syringe filters. The sulfuric acid at 0.05 mM (v/v) was used as the solvent of the HPLC (Shımadzu, Prominence LC-20 A, Kyoto, Japan) device. The HPLC (Shimadzu, Prominence LC-20 A, Kyoto, Japan) operating settings were described as follows: (a) an injection volume of 20 µl, (b) a column temperature of 40 °C, (c) the flow rate was 0.8 ml/min, and the emission wavelength was 210 nm. Ascorbic acid contents in samples were identified based on their retention time, and the peaks were compared to standards. The results were expressed as mg/100 g fresh weight.

### Macro and microelement analysis

Macro and microelement analyses included phosphorus, potassium, magnesium, calcium, iron, manganese, copper, and zinc. The harvested microgreens were washed with distilled water, dried at 65 °C, ground to 20 mesh, and incinerated at 550 °C for 8 h, and the ash dissolved in 3.3% HCl (v/v). Potassium, calcium, and magnesium were measured in emission mode, while iron, manganese, zinc, and copper were measured in absorbance mode using a Varian FS 220 atomic absorption spectrometer (Mulgrave, Virginia, Australia)^15^. Phosphorus was analyzed spectrophotometrically using the Barton method^15^. The phosphorus content in microgreens is determined using a colorimetric method. After dry ashing and dissolving the ash in 3% HCl (v/v), the phosphorus ions react with vanadate and molybdate ions, forming a yellow-colored vanadomolybdo-phosphoric acid complex. The intensity of the yellow color is then measured spectrophotometrically at 420 nm.

### Determination of sugar

**Fig. 2:**
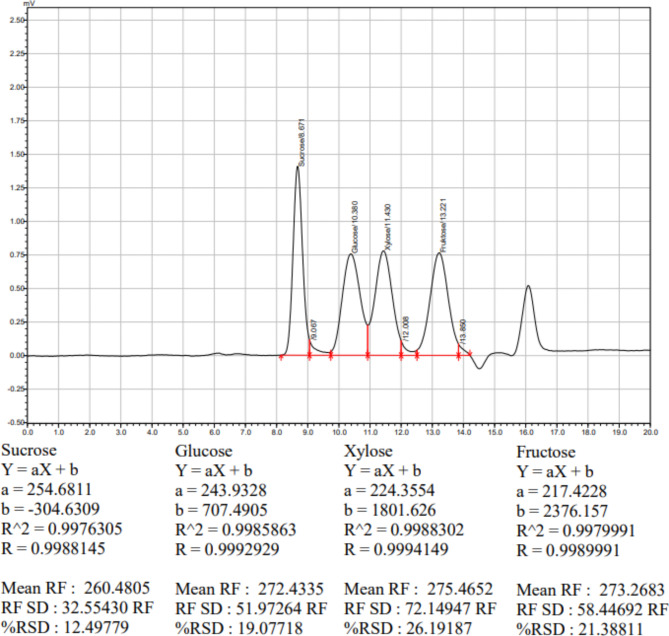
Chromatograms of sugar standards.

Changes in fructose, glucose, and sucrose in microgreen samples were analyzed using HPLC (Shimadzu, Prominence LC-20 A, Kyoto, Japan) with an RID detector and a Shim-Pack HRC NH2 column (300 × 7.8 mm, 5 μm), following Miron and Schaffer’s method^16^. Fructose, glucose, and sucrose are external standards (15-2500 ppm). Each 0.5 g sample was mixed with 2 ml ultrapure water (Millipore Corp., Bedford, MA, USA), sonicated for 15 min, and centrifuged at 5500 rpm for 15 min. The supernatant was filtered and injected into the HPLC for separation at 70 °C with a 0.6 ml/min flow rate using milli-Q water for elution. Sugars were quantified using standards and reported as a percentage of fresh weight, determined via standard calibration curves. The equation of the standard curve of sucrose retention time was 8.679 min, glucose retention time was 10.393 min, xylose retention time was 11.424 min, fructose retention time was 13.211 min. The chromatographic determinations of sugar standards are provided in Fig. 2.

#### Determination of organic acids

**Fig. 3:**
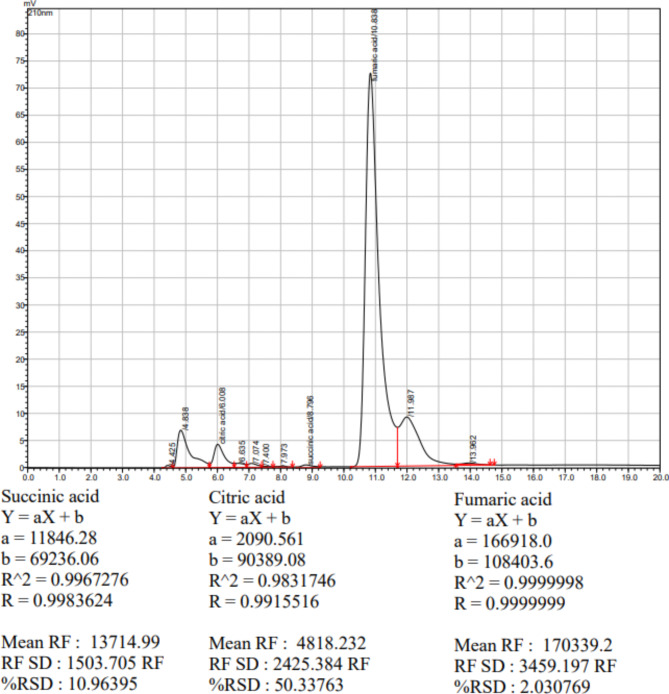
Chromatograms of organic acids standards.

The organic acid contents in microgreen samples were determined following the method of Bozan et al.^14^. Using HPLC (Shimadzu, Prominence LC-20 A, Kyoto, Japan) with a UV detector and an HPX 87 H (300 × 7.8 mm, 5 μm) column, organic acids were extracted by mixing 0.5 g of each sample with 2 ml milli-Q water, followed by sonication for 15 min and centrifugation at 5500 rpm for 15 min. The supernatant was filtered using 0.45 μm Whatman nylon syringe filters. HPLC solvent consisted of 0.05 mM sulfuric acid (v/v). Operating settings included an injection volume of 20 µl, column temperature of 40 °C, flow rate of 0.8 ml/min, and emission wavelength of 210 nm. Organic acid contents were identified based on retention time, compared to standards, and expressed as mg/100 g (w/w) fresh weight. The equation of the standard curve of citric acid retention time was 5.970 min, succinic acid retention time was 8.794 min, and fumaric acid retention time was 10.843 min. The chromatographic determinations of the organic acids standards are provided in Fig. 3.

### Determination of total phenols

Total phenolic content was determined using a spectrophotometric method modified by Spanos and Wrolstad^17^. Each sample, dissolved in 80% methanol (v/v), was subjected to ultrasound for 10 min at 40 °C to enhance phenolic compound liberation, followed by centrifugation at 1500 rpm for 5 min. The supernatant was then mixed with Folin-Ciocalteu reagent and 20% sodium carbonate, incubated in the dark for two h, and the absorbance was read at 765 nm using a UV-1700 PharmoSpec Shimadzu spectrophotometer (Nagoya, Japan). A calibration curve with known concentrations of gallic acid (GA) (0.1-1 mg/ml w/v) was used to determine total phenolic contents, expressed as milligrams of GA equivalents per gram fresh weight (FW). The equation of the standard curve was y = 1.58x + 0.0314; R^2^ = 0.99.

### Determination of total flavonoids

Total flavonoid content was determined according to the method developed by Quettier et al.^18^. Concisely, an aliquot of 1 ml of each extract was mixed with 0.05 ml of AlCl3 (10% w/v), 0.05 ml of potassium acetate (1 M w/v), and 1.8 ml of milli Q-water. A vortex thoroughly mixed all reagents, and they were set in the dark for 45 min at room temperature. After incubation, the absorbance was recorded using a spectrophotometer (UV-1700 PharmoSpec Shimadzu, Nagoya, Japan) at 450 nm. A standard curve of rutin (0.1-1 mg/ml w/v) was prepared in the same conditions of the samples with y = 1.55x + 0.0218 and R^2^ = 0.99 The results are expressed as mg rutin equivalent per 100 g fresh matter.

### Determination of DPPH scavenging activity (%)

Microgreens’ 1,1-diphenyl-2-picrylhydrazyl (DPPH) scavenging activities were determined following the method of Brand-Willials et al.^19^, using a spectrophotometer (UV-1700 PharmoSpec Shimadzu, Nagoya, Japan). Each sample (100 µL) was thoroughly mixed with 3900 µL of DPPH solution (0.06 µM) freshly prepared in 80% methanol. Subsequently, all mixtures were set in the dark for 5 min, and their absorbance was read at 515 nm against a blank. The results were calculated using the equation below:

$$\:DPPH\:\left(\%\right)=\frac{{A}_{c}-{A}_{S}}{{A}_{c}}\times\:100$$

Ac: the absorbance of the control; As: the absorbance of the sample.

### Analysis of volatile aromatic compounds using HS-SPME GC/MS technique

Volatile compounds in microgreens were determined using the Head Space Solid Phase Micro Extraction (HS-SPME) method coupled with Gas Chromatography-Mass Spectrometry (GC/MS Shimadzu GC-2010, North America, USA). Aroma analyses were conducted according to the technique developed by Shalit et al.^20^. From each sample, 500 mg was weighed, mixed with 0.5 mL of CaCl₂, and placed in a headspace vial. Mixtures were incubated for 30 min in a water bath at 40 °C. Then, samples were analyzed on GC-MS with a polar column (HP-Innowax; 60 m × 0.25 mm, 0.25 μm: Length, diameter, particle size) for 70 min. Identification procedures were carried out using Wiley and NIST (National Institute of Standards of Technology) Library Search Software version 11 by comparing the retention times of reference compounds in published sources.

### Nitrate concentration

Nitrate concentration of microgreens was performed colorimetrically using a spectrophotometer (UV-1700 PharmoSpec Shimadzu, Nagoya, Japan) according to Cataldo et al.^21^. Each sample (0.1 g) was added to 10 ml milli-Q water and incubated in a water bath at 45 °C for one h. After centrifugation at 10,000 rpm for 15 min, 100 µl of the upper phase was mixed with 400 µl salicylic acid and incubated in the dark for 20 min. Then, 9.5 ml of 2 N NaOH (w/v) was added to each sample, and absorbance was read at 410 nm. Results are expressed as milligrams per kilogram of fresh weight (FW).

This study complies with relevant institutional, national, and international guidelines and legislation.

### Evaluating data

The data were subjected to variance analysis using the JMP statistical software package (version 7.0, Statistical Software, 2007). Parameters deemed statistically significant at a p-value of less than 0.05 underwent further examination for differences among applications using the Least Significant Difference (LSD) multiple comparison test. Based on this, evaluations and assessments were conducted.

## Results

### Ascorbic acid

The analysis revealed significant variations in ascorbic acid content among the six microgreens. Bean microgreens had the highest level at 80.45 mg/100 g fresh weight, followed by pea microgreens at 70.76 mg/100 g FW and sunflower microgreens at 67.55 mg/100 g FW. In contrast, black radish, red beet, and broccoli microgreens exhibited comparatively lower levels of ascorbic acid, respectively 33.37, 32.72, and 49.02 mg/100 g FW (Table 1).

**Table 1:** Ascorbic acid content in microgreens of six different plant species.

Microgreen species	Microgreens ascorbic acid (mg /100 g FW)
Broccoli	49.02^d^ ± 0.07
Black radish	33.37^e^ ± 0.09
Red beet	32.72^e^ ± 0.08
Pea	70.76^b^ ± 0.01
Sunflower	67.55^c^ ± 0.79
Bean	80.45^a^ ± 0.10
LSD_0.05_	1.4542

FW: Fresh weight. Means with the same letter in the same column indicate no significant differences (*p* < 0.05). *LSD* represents the least significant difference between the means.

### Macro and microelements

The analysis revealed notable variations in the concentrations of macroelements among the six different microgreens (Table 2). Bean microgreens had the highest phosphorus concentration, 4.88 mg/100 g FW, while red beet microgreens had the lowest, 2.57 mg/100 g FW. For potassium, bean microgreens again showed the highest levels, 416.05 mg/100 g FW, while broccoli microgreens had the lowest, 158.88 mg/100 g FW. Broccoli microgreens stood out for magnesium, with the highest concentration, 86.83 mg/100 g FW, while sunflower microgreens had the lowest, 45.96 mg/100 g FW. Sunflower microgreens exhibited the highest calcium concentration, 145.29 mg/100 g FW, while red beet microgreens had the lowest, 67.18 mg/100 g FW (Table 2).

**Table 2:** The macroelements concentrations of the microgreens (mg/100 g FW).

Microgreen species	*P*	K	Mg	Ca
Broccoli	27.7^a^ ± 0.0	158.88^c^ ± 10.9	86.83^a^ ± 12.1	111.83^b^ ± 15.2
Black radish	31.3^a^ ± 0.2	187.07^bc^ ± 23.9	49.07^cd^ ± 7.5	93.94^b^ ± 17.0
Red beet	25.7^a^ ± 0.0	252.70^b^ ± 9.2	49.28^cd^ ± 1.6	67.18^c^ ± 4.8
Pea	46.1^a^ ± 0.1	367.33^a^ ± 9.6	61.36^bc^ ± 13.6	99.07^b^ ± 21.1
Sunflower	35.0^a^ ± 0.1	273.47^b^ ± 8.9	45.96^d^ ± 11.5	148.63^a^ ± 8.1
Bean	48.8^a^ ± 0.1	416.05^a^ ± 6.9	74.15^ab^ ± 4.3	145.29^a^ ± 16.7
LSD_0.05_	23.3928	76.2916	14.9346	19.1197

FW: Fresh weight. Means with the same letter in the same column indicate no significant differences (*p* < 0.05). *LSD* represents the least significant difference between the means.

Broccoli microgreens had the highest iron concentration, 2610.42 µg/100 g FW, and manganese content, 350.56 µg/100 g FW. Pea microgreens exhibited the highest copper concentration, 221.91 µg/100 g FW, while sunflower microgreens had the highest zinc concentration, 956.34 µg/100 g FW. These diverse microelement profiles are summarized in Table 3, illustrating the variability among microgreen varieties.

**Table 3:** The microelements content of microgreens (µg/100 g FW).

Microgreen species	Fe	Mn	Zn	Cu
Broccoli	2610^a^ ± 1.4	350.56^a^ ± 16.5	119.28^b^ ± 5.1	856.00^b^ ± 6.5
Black radish	565^d^ ± 6.4	176.32^b^ ± 18.9	31.92^c^ ± 4.5	458.84^d^ ± 44.9
Red beet	677^c^ ± 4.3	301.05^a^ ± 27.3	96.74^bc^ ± 9.8	486.53^d^ ± 12.5
Pea	1012^b^ ± 7.5	183.73^b^ ± 13.6	221.91^a^ ± 25.0	879.97^ab^ ± 13.6
Sunflower	524^d^ ± 6.7	229.32^ab^ ± 19.0	124.74^b^ ± 5.3	956.34^a^ ± 7.4
Bean	1054^b^ ± 7.7	222.39^ab^ ± 17.3	129.78^b^ ± 1.7	705.88^c^ ± 1.00
LSD_0.05_	116.4950	116.2218	64.2351	127.9268

FW: Fresh weight. Means with the same letter in the same column indicate no significant differences (*p* < 0.05). *LSD* represents the least significant difference between the means.

### Sugar concentration

Sugar levels in microgreens, primarily consisting of glucose, fructose, and sucrose, are relatively low. Sucrose levels range from 0.013 to 0.248 mg/100 g FW, glucose levels from 0.114 to 0.580 mg/100 g FW, and fructose levels from 0.074 to 0.671 mg/100 g FW (Table 4). Among the six microgreens, red beet presented the highest amount of sugar. These results emphasize the modest sugar content found in microgreens.

**Table 4:** Sugar contents of six different microgreens (mg /100 g FW).

Microgreen species	Sucrose	Glucose	Fructose
Broccoli	0.019^c^ ± 0.01	0.114^d^ ± 0.01	0.074^d^ ± 0.02
Black radish	0.054^b^ ± 0.01	0.422^bc^ ± 0.11	0.210^c^ ± 0.01
Red beet	0.248^a^ ± 0.01	0.580^a^ ± 0.01	0.671^a^ ± 0.00
Pea	0.013^c^ ± 0.01	0.502^ab^ ± 0.01	0.090^d^ ± 0.01
Sunflower	0.040^b^ ± 0.01	0.502^ab^ ± 0.07	0.174^c^ ± 0.02
Bean	0.016^c^ ± 0.01	0.315^c^ ± 0.07	0.451^b^ ± 0.02
LSD_0.05_	0.0205	0.1212	0.0554

FW: Fresh weight. Means with the same letter in the same column indicate no significant differences (*p* < 0.05). *LSD* represents the least significant difference between the means.

### Organic acid concentration

**Fig. 4:**
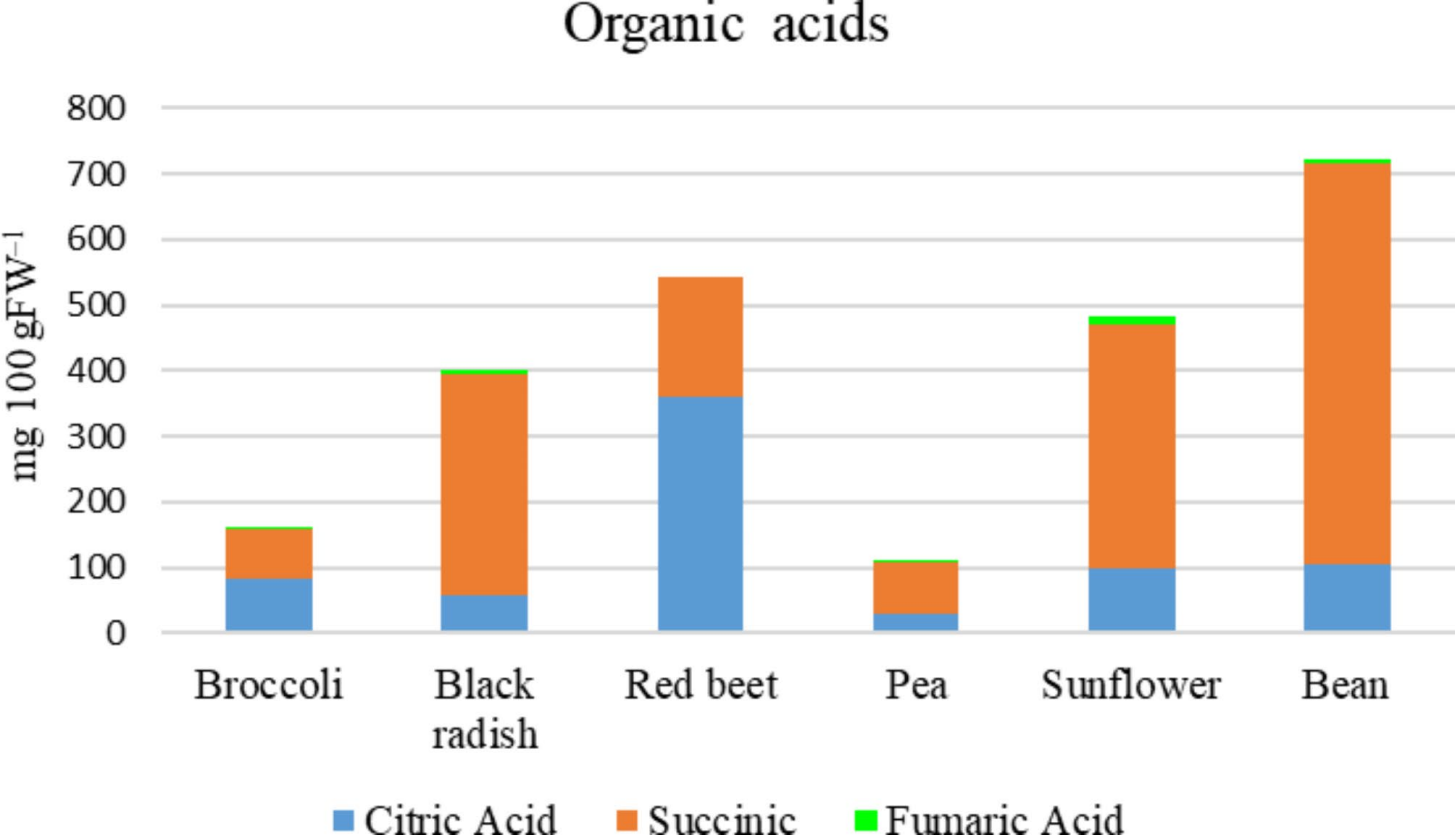
Organic acid contents of six different microgreens.

Red beet microgreens had the highest concentration of citric acid at 358.83 mg/100 g, followed by beans at 103.90 mg/100 g and sunflower microgreens at 99.41 mg/100 g. Succinic acid levels followed a similar trend, with bean microgreens registering the highest concentration at 611.99 mg/100 g and sunflower microgreens at 370.68 mg/100 g. Fumaric acid levels were highest in sunflower microgreens at 12.56 mg/100 g, followed by beans at 5.75 mg/100 g (Fig. 4). These findings highlight the diverse organic acid profiles among different microgreens.

### Total phenol concentration

Broccoli microgreens show the highest total phenolic content among all species at 825.53 mg GAE/100 g FW (Table 5), closely followed by black radish microgreens at 659.53 mg GAE/100 g FW. Sunflower microgreens also have a high total phenolic content at 638.94 mg GAE/100 g FW, comparable to broccoli and black radish. Pea microgreens exhibit a slightly lower total phenolic content of 538.74 mg GAE/100 g FW than broccoli, black radish, and sunflower. Bean microgreens have a total phenolic content of 413.70 mg GAE/100 g FW, lower than broccoli, black radish, sunflower, and pea microgreens. Red beet microgreens have the lowest total phenolic content among the group at 373.90 mg GAE/100 g FW.

### Total flavonoid concentration

Red beet microgreens have the highest total flavonoid content among the group at 1625 mg/100 g FW, followed closely by black radish microgreens at 1193 mg/100 g FW. Bean microgreens also contain significant flavonoids, totaling 758 mg/100 g FW. Broccoli microgreens exhibit a moderate level of flavonoid content, 707 mg/100 g FW. Pea microgreens have a relatively lower total flavonoid content than others in the experiment, with 482 mg/100 g FW. Sunflower microgreens have the lowest total flavonoid content, 217 mg/100 g FW (Table 5).

### DPPH scavenging capacity

As Table 5 depicts, all microgreens exhibited a high antioxidant capacity to trap free DPPH radicals. Black radish possessed the highest DPPH scavenging power, 83.32%, followed by bean, sunflower, red beet, and broccoli. Pea microgreens exhibited the lowest antioxidant activity of 70.13%.

### Nitrate concentration

Red beet microgreens have the highest nitrate content among the group at 636.41 mg/kg FW (Table 5), followed by black radish microgreens at 391.75 mg/kg FW. Bean microgreens have a moderate nitrate content at 242.65 mg/kg FW, while broccoli microgreens exhibit a relatively lower content at 149.09 mg/kg FW. Sunflower microgreens have a low nitrate content of 77.25 mg/kg FW, the least among the microgreens. Pea microgreens did not contain detectable levels of nitrates.

**Table 5:** Total phenol, total flavonoid, antioxidant, and nitrate contents of the microgreens.

Microgreen species	TPC (mg GA/100 g FW)	TFC (mg RU/100 g FW)	DPPH (%)	Nitrate (mg/kg FW)
Broccoli	825.53^a^ ± 6.30	707^d^ ± 0.21	75.98^c^ ± 0.01	149.09^cd^ ± 3.69
Black radish	659.53^b^ ± 19.29	1193^b^ ± 0.65	83.32^a^ ± 0.19	391.75^b^ ± 46.98
Red beet	373.90^d^ ± 37.95	1625^a^ ± 0.35	77.31^bc^ ± 2.00	636.41^a^ ± 52.78
Pea	538.74^c^ ± 43.37	482^e^ ± 0.64	70.13^d^ ± 4.77	Not detected
Sunflower	638.94^b^ ± 46.14	217^f^ ± 0.11	81.51^ab^ ± 0.50	77.25^de^ ± 10.12
Bean	413.70^d^ ± 58.56	758^c^ ± 0.36	82.76^a^ ± 0.21	242.65^c^ ± 25.36
LSD_0.05_	59.6338	0.8079	5.3189	134.7758

TPC: Total phenol content, TFC: total flavonoid content, GA: Gallic acid, RU: Rutin, DPPH: 2.2-difenil-1-pikrilhidrazil, FW: Fresh weight. Means with the same letter in the same column indicate no significant differences (*p* < 0.05). *LSD* represents the least significant difference between the means.

### Volatile aromatic compounds

**Fig. 5:**
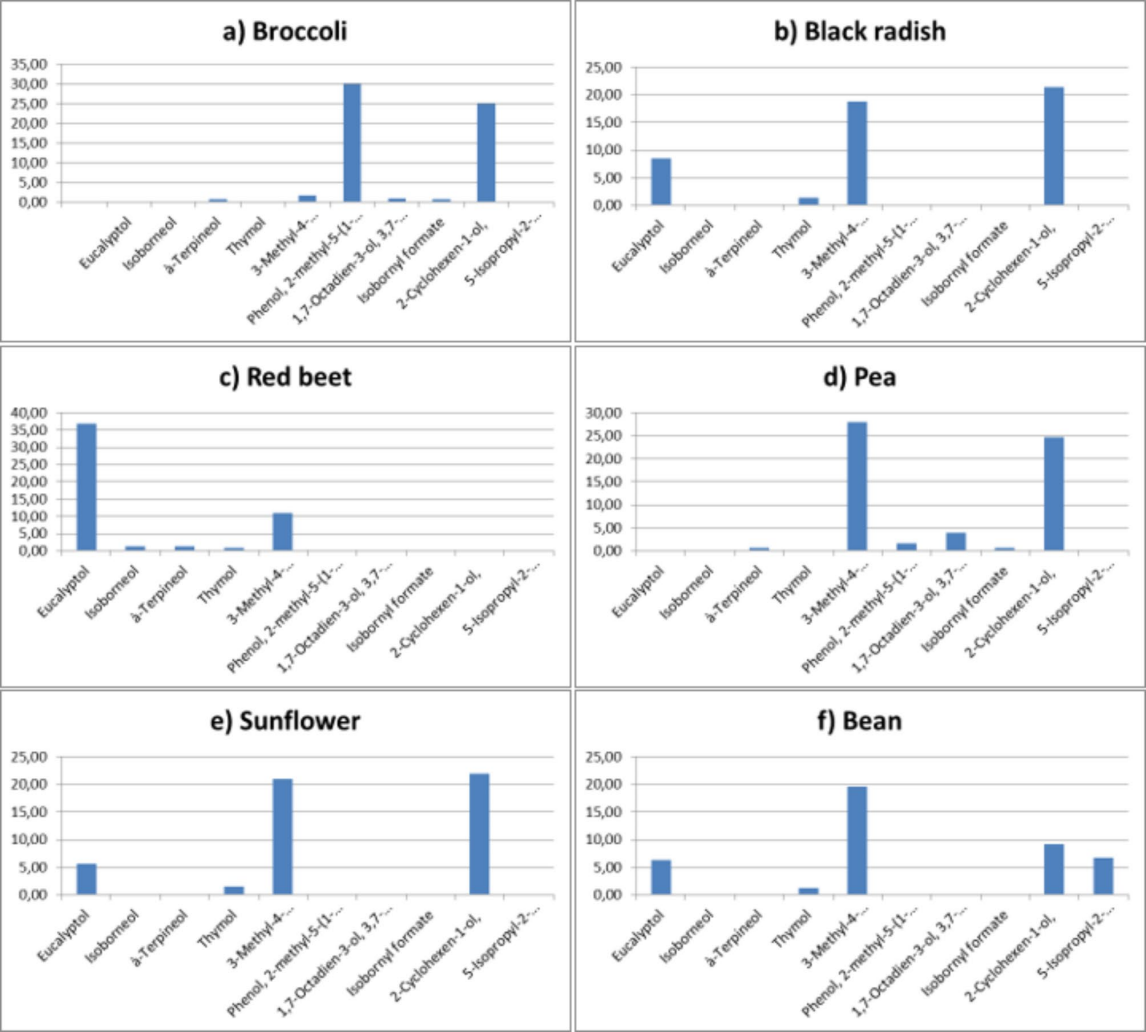
The alcohol profile of microgreens from six different species.

**Fig. 6:**
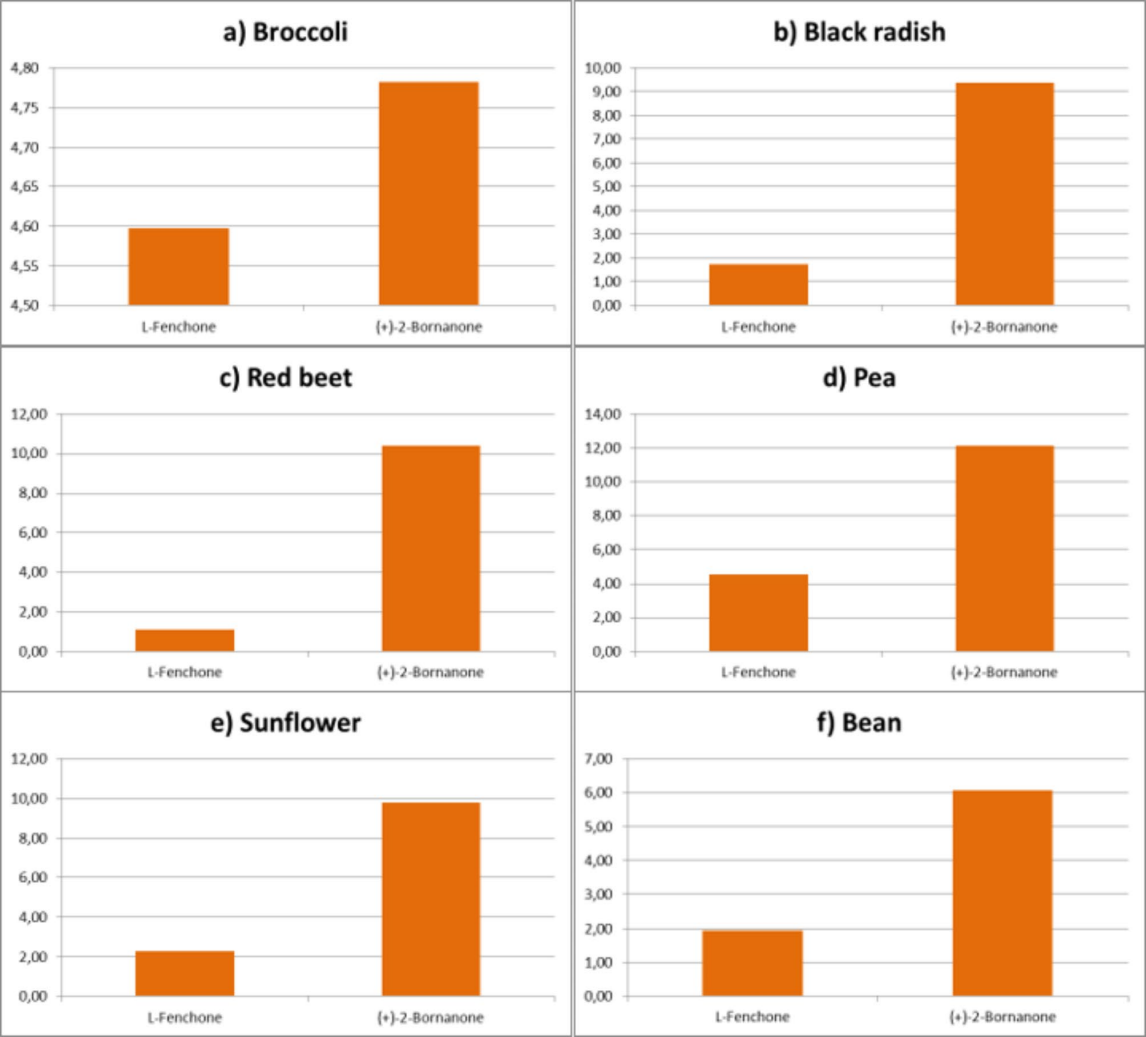
The ketones profile of microgreens from six different species.

**Fig. 7:**
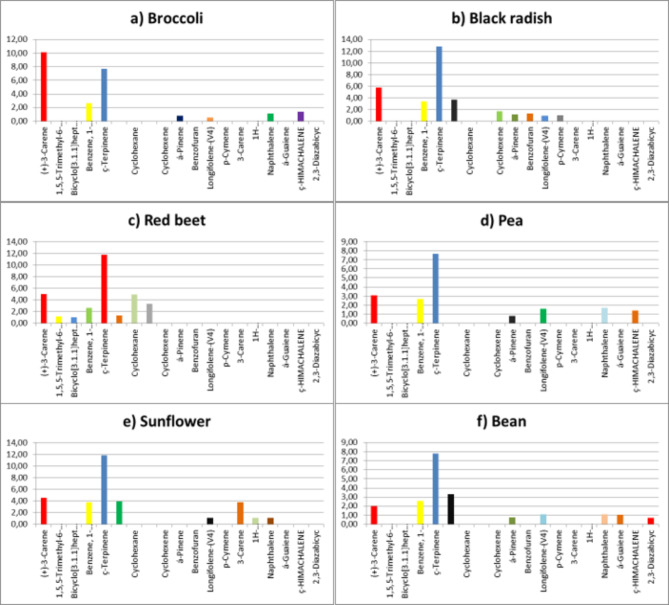
The terpene profile of microgreens from six different species.

In broccoli microgreens, fifteen aroma compounds were identified and categorized into groups: six alcohols, two ketones, and seven terpenes. Alcohols constituted 58.81% of the aroma compounds, with phenol (29.92%) and cyclohexene (24.95%) predominant. Ketones accounted for 9.38% of the total, with (+)-2-bornanone (4.78%) and L-fenchone (4.60%) as significant contributors. Terpenes represented 24.32% of the aroma compounds, primarily composed of (+)-3-carene (10.13%) and α-terpinene (7.71%) (Figs. 5a, 6a and 7a).

Black radish microgreens contained sixteen identified aroma compounds, categorized into four alcohols, one aldehyde, two ketones, and nine terpenes. Alcohols constituted 49.88%, with 2-cyclohexene (21.34%) and 3-methyl-4-isopropylphenol (18.75%) prominent. Ketones accounted for 11.11%, while terpenes constituted 31.81%, with α-terpinene (12.83%) and carene (5.75%) as major components (Figs. 5b, 6b and 7b).

Red beet microgreens exhibited fifteen aroma compounds, categorized into 5 alcohols, 2 ketones, and 8 terpenes. Alcohols constituted 36.94%, with eucalyptol (36.94%) and 3-methyl-4-isopropylphenol (10.94%) predominant. Ketones accounted for 10.41%, while terpenes represented 4.97%, with α-terpinone (11.75%) and carene (4.97%) notable (Figs. 5c, 6c and 7c).

Pea microgreens contained fifteen aroma compounds, including 6 alcohols, 2 ketones, and 7 terpenes. Alcohols constituted 59.75%, with 3-methyl-4-isopropylphenol (27.96%) and 2-cyclohexene (24.76%) as predominant. Ketones accounted for 16.70%, while terpenes represented 18.83%, with α-terpinene (7.65%) notable (Figs. 5d, 6d and 7d).

Sunflower microgreens contained fourteen aroma compounds, comprising 4 alcohols, 2 ketones, and 8 terpenes. Alcohols constituted 50.05%, with 2-cyclohexene (21.92%) and 3-methyl-4-isopropylphenol (21.02%) as predominant. Ketones accounted for 12.04%, while terpenes represented 31.04%, with α-terpinene (11.85%) notable (Figs. 5e, 6e and 7e).

Bean microgreens contained sixteen aroma compounds, classified into 5 alcohols, 2 ketones, and 9 terpenes. Alcohols constituted 43.09%, with 3-methyl-4-isopropylphenol (19.62%) and cyclohexene (9.23%) being notable. Ketones accounted for 8.01%, while terpenes represented 20.29%, with α-terpinone (7.80%) being notable (Figs. 5f, 6f and 7f).

## Discussion

In this study, we observed significant variations in the bioactive composition among the six microgreens, with some species excelling in specific nutrients and bioactive compounds. For instance, bean microgreens had the highest ascorbic acid content (80.45 mg/100 g FW), while broccoli microgreens exhibited the most significant total phenolic content (825.53 mg GA/100 g FW). These findings highlight the species-dependent nature of nutrient accumulation in microgreens, likely influenced by genetic factors and nutrient uptake efficiency^22–24^.

One of the key trends observed was the strong antioxidant capacity across all microgreen species, with black radish showing the highest DPPH scavenging activity (83.32%). This can be attributed to the elevated levels of phenolic and flavonoid compounds, particularly in black radish and red beet microgreens, which contribute to antioxidant activity. The phenolic content of broccoli microgreens also underscores their potential as a functional food with health-promoting benefits, aligning with prior research on *Brassicaceae* family members^5,25,26^. The DPPH capacity determined in the six plant species in our study, ranging from 70.13 to 83.32%, was found to be higher than the values reported by Kowitcharoen et al.^27^ for broccoli, radish, fenugreek, green pea, mung bean, roselle, black sesame, Chinese kale, purple radish, red cabbage, and lentil microgreens, except for buckwheat.

When comparing the flavonoid content, red beet microgreens stood out with the highest levels (1625 mg/100 g FW), followed by black radish (1193 mg/100 g FW). These results suggest that red beet microgreens may serve as a potent source of flavonoids, associated with various health benefits, including anti-inflammatory and anti-carcinogenic properties. The flavonoid profiles of the microgreens, in conjunction with their antioxidant capacities, further reinforce their potential as a dietary intervention to combat oxidative stress^5,22^.

The variability in organic acid contents also revealed essential relationships. Red beet microgreens had the highest citric acid content, while bean microgreens excelled in succinic acid. These organic acids play critical roles in plant metabolism and human health, potentially influencing these microgreens’ flavor profile and metabolic benefits^23^.

In our study, red beet microgreens were identified as the most abundant species for sugar content. The glucose, fructose, and sucrose content was higher than that reported for beet microgreens by Wojdyło et al.^28^.

Our findings emphasize microgreens’ nutrient density and bioactive potential, with notable variations among species in their mineral, phenolic, flavonoid, and antioxidant capacities. These trends reflect the complex interactions between genetic and environmental factors governing microgreens’ nutrient accumulation. The distinct nutrient profiles observed in our study suggest that specific microgreen species could be strategically incorporated into diets to address particular health needs, such as enhancing antioxidant intake or boosting flavonoid consumption^22,25,26^.

In our study, the ascorbic acid content was highest in bean microgreens (80.45 mg/100 g FW). When compared to the results reported by Kowitcharoen et al.^27^, it is higher than those for broccoli, radish, fenugreek, green pea, mung bean, buckwheat, roselle, and black sesame microgreens. However, it is lower than those for Chinese kale, purple radish, red cabbage, and lentil microgreens. Our ascorbic acid findings are consistent with the data reported by Altuner for five legumes and seven cereals^29^. Ascorbic acid acts as an enzyme cofactor involved in the regulation of photosynthesis. This vitamin is a potent antioxidant required for several biological processes, including collagen production and immune system regulation^25^. Khoja et al.^30^ reported that ascorbic acid and iron absorption enhancers may be found in greater concentrations in microgreens. Twenty-five commercially available microgreens of various vegetables contained more vitamins and carotenoids than their mature plant counterparts^8^. Martínez-Ispizua et al.^22^ reported that microgreens and baby leaves could contain significantly higher levels of vitamins, minerals, antioxidant properties such as phenols and ascorbic acid, and other health-beneficial phytonutrients compared to mature leaves. For these reasons, these tiny plants are now recognized as functional foods^23^.

However, the study of Kowitcharoen et al.^27^ showed that broccoli and radishes present higher ascorbic acid content than our study and a lower content in beans and peas. Ghoora, Haldipur, and Srividya^31^ reported that sunflowers exhibit a higher ascorbic acid than our findings. These data reinforce the idea that environmental conditions play a clear role in microgreen’s bioactive contents.

Apart from ascorbic acid content, we observed a difference in mineral contents among all microgreens, which can be explained by the variation in the expression of nutrient transporter genes that enhance nutrient uptake for each microgreen^32^. Several studies demonstrate that microgreens are an excellent source of mineral cofactors for antioxidant enzymes, such as Zn, Se, and Cu^24^. Consequently, they are known as antioxidant minerals since they support the body’s defense mechanism.

Additionally, microgreens contain a wealth of phytochemicals that can improve human health, such as phenolic compounds. Xiao et al.^33^ reported a total phenolic content of 283 mg GAE/100 g FW for broccoli. In our study, broccoli recorded the highest TPC at 826 mg GAE/100 g FW. In the same study by Xiao et al.^33^, the TPC content of red cabbage was reported as 307 mg GAE/100 g FW, while in our study, it was found to be 374 mg GAE/100 g FW. These differences may be attributed to genetic diversity and growing conditions within the same species. The findings of Kowitcharoen et al.^27^ reveal that broccoli (87.56 mg GAE 100 g^− 1^), radish (132.78-145.04 mg GAE 100 g^− 1^), pea (38.14 mg GAE 100 g^− 1^), and bean (59.95 mg GAE 100 g^− 1^) presented a lower content in phenolic compounds than our study. However, our findings follow those of Marchioni et al.^26^, where broccoli exhibited the highest levels of phenolic compounds. These data are supported by the report of Ebert^5^, who revealed that the principal bioactive compounds in the *Brassicaceae* family are phenolic compounds, which have been widely investigated due to their recognized health-promoting capacities. The high phenolic compounds and flavonoids enhance the antioxidant capacity of microgreen species. Their potent antioxidant capacity is linked to their ability to chelate metal ions, neutralize free radicals, donate electrons to oxidized species, and attenuate the production of reactive oxygen species (ROS) by enhancing antioxidant enzyme activity or blocking enzymes that promote pro-oxidant effects^34^.

Here, the microgreens’ antioxidant power was evaluated by the capability of each species to trap DPPH free radicals by donating a hydrogen atom and converting the radical to a reduced form^27^. The DPPH capacity determined in the six plant species in our study, ranging from 70.13 to 83.32%, was found to be higher than reported by Kowitcharoen et al.^27^ for broccoli, radish, fenugreek, green pea, mung bean, roselle, black sesame, Chinese kale, purple radish, red cabbage, and lentil microgreens, except for buckwheat.

Prior research^35,36^ showed that external factors, such as soil composition, application of fertilizers, water availability, environmental conditions, and maturity stage, were the primary sources of variability in nutrient composition. In addition to the intrinsic variables that affect microgreen species and varieties. The primary factor influencing mineral content in our study was the plant maturity stage because the grower maintained the same levels of all other extrinsic parameters. However, the current experiment indicates that not all minerals showed the same pattern. The tendency varied depending on the crop. Our study used a uniform nutrient solution across all six microgreen species to evaluate their mineral content. It is important to note that while the same nutrient solution was applied to all plant types, the variations in nutrient uptake capacities among the different microgreen species led to differential effects on their macro and micro-mineral contents^32^. This discrepancy underscores the influence of species-specific nutrient absorption and accumulation characteristics, which resulted in varied mineral profiles across the microgreens. Thus, the interaction between the nutrient solution and the inherent capabilities of each plant species played a crucial role in determining the final mineral content observed in this study^24^.

Microgreens have a higher mineral bioavailability than mature vegetables because they are ingested very early when epidermal growth is low. In this situation, microgreens could be a functional meal^37^. In their study on thirty different *Brassicaceae* microgreens, Xiao et al.^36^ reported macro elements (mg/100 g FW) as Ca ranging from 41 to 98, Mg 31–66, P 52–86, and K 176–387. The microelements were reported as Fe 0.47–0.84, Mn 0.17–0.48, Zn 0.22–0.51, and Cu 0.04–0.12 (mg/100 g FW). These values are consistent with our study’s macro and microelements. Differences can be attributed to genetic and environmental factors.

Plant species and even cultivars of the same species exhibit significant variation in the nitrate concentration, as do genotypes with varying ploidy. Lenzi et al.^38^ found that nitrate concentration in microgreens was significantly lower than in baby greens. Factors contributing to variations in nitrate accumulation may involve genetic diversity, enzymatic processes such as nitrate and nitrite reductase activity, alterations in nitrate uptake, availability of cofactors essential for enzyme function, and/or fluctuations in the production of electron donors crucial for the assimilation pathway. Pinto et al.^39^ assert that numerous factors, including NO_3_-phytoavailability, temperature, water availability, light intensity, nitrate reductase activity, and harvest time, can contribute to NO_3_-accumulation in plants. It is hypothesized that NO_3_- accumulation in mature plants is mainly attributed to elevated soil nitrogen dioxide phytoavailability and the restricted capacity of nitrogen reductase to reduce the anion during later stages of plant growth. This is assumed while maintaining light intensity, temperature, harvest date, and water availability throughout the study. Moreover, variations in photosynthetic capacity, the capacity to produce and translocate respiratory substrate and reducing equivalents, or variations in the ability to translocate the absorbed nitrate to reduction sites can all be linked to differing capacities of nitrate accumulation. As the amount of carbohydrate in the vacuoles increases, nitrate accumulation decreases^27^.

Compared to sucrose content, the high glucose levels in different microgreen species may be explained by the sucrase enzyme’s glycolysis of sucrose into fructose and glucose. Regarding uptake, conversion, growth, and respiration, many plant cells react differently to fructose and glucose, even though they have identical chemical structures and are highly interconvertible in glycolysis. Distinct growth efficiencies for glucose and fructose were observed in cell suspensions sourced from various plant species^40^. Along with the variation in sugar contents, different species of microgreens have a variability in organic acid contents. Roots regulate organic acids produced in the rhizosphere. The difference in organic acid amounts may be explained by the variation of genetic factors, including differences in individual gene expression patterns and metabolic gene networks^41^.

Besides the richness and variability of bioactive compounds, microgreens showed high aromatic profiles depending on the season and genetic profile^42^. Dimite et al.^43^ demonstrated that the release of volatile compounds is lower in mature plants compared to microgreens. This decline can be attributed to the transition from a juvenile leaf structure, which is more delicate, to an adult one characterized by a higher presence of lignin, a thicker cuticle and epidermis, and enhanced stomatal regulation. Balik et al.^44^ found that broccoli in peat-based medium had the highest total phenolic content, while red beet in agril had the highest flavonoid content. Black radish showed the highest vitamin C in agril and protein in filter paper. The highest SPAD–chlorophyll values were recorded in black radish grown in agril. Microgreens grown in vermicompost and a peat-based medium had the highest yields. These findings suggest that while optimal conditions vary by species, the choice of growing medium plays a crucial role in determining microgreens’ quality and nutrient content.

### Conclusions

The findings of this research confirm that microgreens possess an exceptional nutritional profile, with each species exhibiting unique benefits. Red beet microgreens, for instance, were richest in organic acids, particularly citric acid and flavonoids, supporting antioxidant activity and potential anti-inflammatory effects. In contrast, black radish microgreens showed the highest DPPH antioxidant capacity and phenolic content, indicating potent free radical neutralization. Bean microgreens stood out with their high ascorbic acid content, essential for immune health and skin maintenance. Furthermore, sunflower microgreens had the highest calcium and fumaric acid levels, indicating benefits for bone health and energy production. Broccoli microgreens were richest in phenolic compounds and contained high iron and manganese levels, both critical for red blood cell production. Finally, pea microgreens excelled in phosphorus and copper concentrations, crucial for bone and cardiovascular health. Based on the identified volatile aromatic compounds, the ranking from most to least aromatic was as follows: black radish, pea, sunflower, broccoli, red beet, and bean. Thus, encouraging the widespread production and consumption of microgreens could positively influence consumer health and well-being. Future research should focus on optimizing growing conditions and exploring the potential health benefits of microgreens in addressing specific micronutrient deficiencies.

## Electronic supplementary material

Below is the link to the electronic supplementary material.

Supplementary Material 1

## Data Availability

The data presented in this study are available in the article.
