# Supplementary material for: Nutritional quality profiles of six microgreens
Source: Sci Rep. 2025 Feb 20;15:6213. doi: 10.1038/s41598-025-85860-z (PMC11842852; doi:10.1038/s41598-025-85860-z)
Supplement: Supplementary file 1 — Supplementary Material 1 [file 41598_2025_85860_MOESM1_ESM.docx]

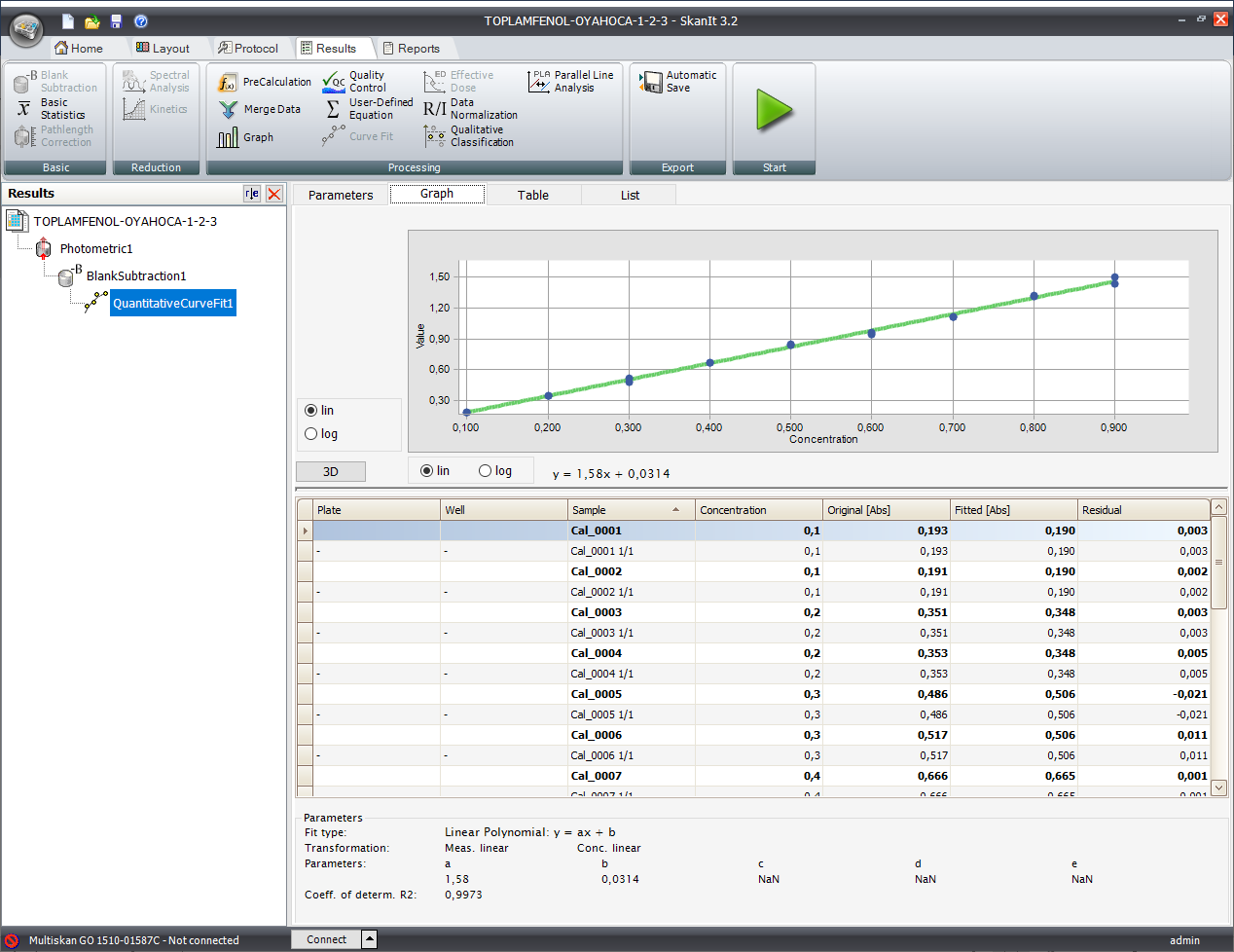


Supplementary figure 1. Total phenols calibration curve and equation fit


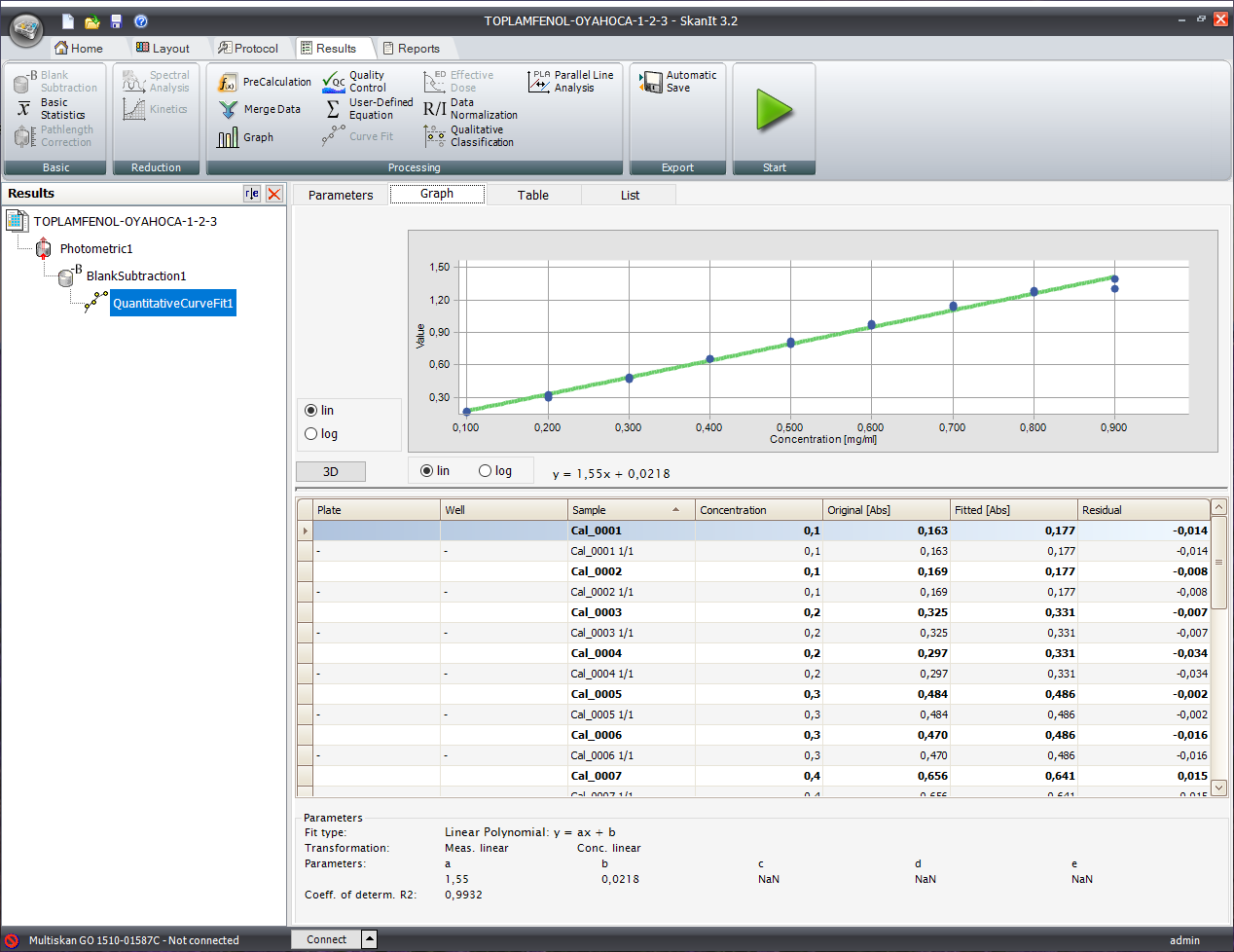


Supplementary figure 2. Total flavonoids calibration curve and equation fit
